# Anti‐dyslipidemic effects of *Pueraria lobata* root and *Glycine max* (*L*.) Merrill extracts fermented with *Lactiplantibacillus plantarum* in ovariectomized mice

**DOI:** 10.1002/fsn3.4356

**Published:** 2024-07-29

**Authors:** Hyo‐Min Jang, Jimyeong Ha, Insuk Choi

**Affiliations:** ^1^ The 2nd Research Institute CMG Pharmaceutical Co., Ltd. Seongnam Korea; ^2^ Center for Consumer Health 1 Research CHA Advanced Research Institute Seongnam Korea

**Keywords:** aglycone, eNOS‐NO‐cGMP signaling pathway, fermentation, *Lactiplantibacillus plantarum*, menopause

## Abstract

*Pueraria lobata* root and *Glycine max* (*L.*) Merrill are rich in phytoestrogens. However, these bioactive ingredients have limited bioavailability due to their high molecular weight. In this study, we extracted two natural products and fermented with *Lactiplantibacillus plantarum* before mixing the fermented extracts (FPE‐FGE). To understand whether FPE‐FGE could alleviate menopause with dyslipidemia, we examined their effects on ovariectomy (OVX)‐induced dyslipidemia in mice. Oral administration of the FPE‐FGE (1:9, 3:7, and 9:1) did not affect safety‐related biomarkers, such as uterus index (%), vagina index (%), aspartate aminotransferase, alanine aminotransferase, blood urea nitrogen, and creatinine. Furthermore, FPE‐FGE (1:9, 3:7, and 9:1) increased the levels of 17β‐estradiol (E2) and expression of uterus estrogen receptor β (ERβ); there was little effect on the expression of uterus estrogen receptor α (ERα), and reduced the levels of gonadotropins, such as luteinizing hormone (LH) and follicle‐stimulating hormone (FSH). However, only the FPE‐FGE (3:7) reduced the levels of blood lipids, including total cholesterol (TC) and LDL‐cholesterol (LDL‐C). Accordingly, FPE‐FGE (3:7) upregulated endothelial nitric oxide synthase (eNOS), nitric oxide (NO), cyclic guanosine monophosphate (cGMP), and protein kinase G (PKG). In conclusion, FPE‐FGE (3:7) attenuated the menopausal dyslipidemia by upregulating eNOS‐NO‐cGMP signaling pathway.

## Graphical abstract

FPE‐FGE (3:7), which includes a combination of various glycosides, can reduce the levels of blood lipids by upregulating the eNOS‐NO‐cGMP signaling pathway.

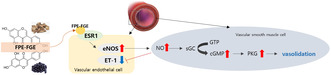

## 1 INTRODUCTION

Cardiovascular disease is the leading cause of mortality among women worldwide (Chang et al., 2011; Garcia et al., 2016). In particular, the cardiovascular disease risk significantly increases in women experiencing menopause due to dyslipidemia (Anagnostis et al., 2022; Wild et al., 2021). Menopause has been approximated in experimental female rodents by treating them with ovariectomy (OVX) surgery (Souza et al., 2019), which induces the reduction of 17β‐estradiol (E2) and stimulates luteinizing hormone (LH) and follicle‐stimulating hormone (FSH) production (Kim et al., 2014; Luderer & Schwartz, 1994). However, increased LH suppresses the endothelial nitric oxide synthase (eNOS)‐nitric oxide (NO)‐cyclic guanosine monophosphate (cGMP) signaling pathway, resulting in lipid accumulation (Meng et al., 2019), and FSH induces dyslipidemia through inhibiting hepatic cholesterol metabolism (Song et al., 2016). In contrast, E2 induces eNOS and NO release, activating the eNOS‐NO‐cGMP signaling pathway (Vera‐Arzave et al., 2019). In addition, E2 regulates the secretion of LH and FSH through the negative feedback mechanism (Shaw et al., 2010).

Furthermore, NO is an essential vasodilator produced by the vascular endothelium via eNOS (Lamping et al., 2000; Manicam et al., 2017), and diffuses from the vascular endothelium to the smooth muscle cell, activating soluble guanylyl cyclase (sGC); this results in increased cGMP production. Subsequently, the cGMP activates protein kinase G (PKG), resulting in vasodilation (Bucci et al., 2012; Chen et al., 2023); this leading to vasodilation‐mediated anti‐atherosclerotic effects by improving vasomotor dysfunction (Raman et al., 2011; Tentolouris et al., 2000). Therefore, regulating the eNOS‐NO‐cGMP signaling pathway is a beneficial strategy for treating menopause‐induced dyslipidemia in women.

*Pueraria lobata* roots and *Glycine max* (*L*.) Merrill are rich in phytoestrogens, such as the isoflavone glycosides puerarin, daidzin, and genistin (Choi et al., 2020; Wagle et al., 2019). Isoflavone glycosides exert estrogenic effects in women experiencing menopause as their chemical structure is similar to estrogen, lowering blood cholesterol (Shidfar et al., 2009; Taku et al., 2007). However, these bioactive ingredients have a high molecular weight, so the absorption rate in the body is poor (Piskula et al., 1999; Zhang et al., 2011). In recent years, isoflavone glycosides have been fermented to reduce glucose, resulting in an increased absorption rate in the body (Nagino et al., 2016; Okabe et al., 2011).

Therefore, in a previous study, we extracted two natural products and fermented with *Lactiplantibacillus plantarum* before mixing the fermented extracts (FPE‐FGE) and checked the expression of estrogen receptors in cells treated with FPE‐FGE (1:9, 3:7, 5:5, and 9:1) to determine the best ratio. The FPE‐FGE (3:7) was the most effective because upon treatment with this mixture, the levels of ESR1 were lower and those of ERβ were higher than those in the E2 group (Ha et al., 2023). We also found that the FPE‐FGE (3:7) additively or synergistically ameliorates the depressive‐like behavior of ovariectomized mice by upregulating the hippocampal brain‐derived neurotrophic factor (Ha et al., 2023). However, the fermented extract mixture's anti‐dyslipidemic effects have not been studied in vivo.

In this study, to study the anti‐dyslipidemic effects of FPE‐FGE (3:7), we extracted *Pueraria lobata* roots and *Glycine max* (*L*.) Merrill and fermented with *L. plantarum* before being freeze‐dried and combined in differing ration to further investigated the dyslipidemia‐related biomarkers in OVX mice.

## 2 MATERIALS AND METHODS

### 2.1 Fermentation of *Pueraria lobata* root and *Glycine max* (*L.*) Merrill

Fifty‐six LAB strains were isolated from Korean traditional fermented food “kimchi” and identified, as previously reported (Ha et al., 2023). The isolated bacteria were cultured in the general lactic acid bacteria (LAB) De Man, Rogosa, and Sharpe broth (Becton, Dickinson and Company, Radnor, PA, USA) and used for the fermentation of *Pueraria lobata* root or *Glycine max* (*L*.) Merrill extracts. The fermented *Pueraria lobata* root and *Glycine max* (*L*.) Merrill extracts were freeze‐dried for future use.

### 2.2 High‐performance liquid chromatography (HPLC) analysis

The analysis was performed using an Agilent Technologies 1200 Series HPLC system (Agilent Technologies, Santa Clara, CA, USA). A C18 column (Phenomonex Kinetex, CA, USA) was used to analyze all samples. The mobile phases were water (solvent A) and acetonitrile with 0.1% acetic acid (solvent B) in the gradient mode (Ha et al., 2023).

### 2.3 Animals

Female Institute of Cancer Research (ICR) mice (6 weeks old; 20 ± 2 g) were purchased from JA BIO, Inc. (Suwon, Korea). Mice were kept in wire cages under ventilated conditions (20–20°C, 50 ± 10% relative humidity, and 12 h light–dark cycle) and fed commercial standard laboratory mouse chow and water ad libitum.

All experiments were performed following the National Institute of Health (NIH) and CHA Bio Complex guidelines. All animal experiments were approved by the Institutional Animal Care and Use Committee of the CHA Bio Complex (IACUC No. 220163).

### 2.4 Preparation of mice with menopause and dyslipidemia

After acclimation, all the mice, except those in the sham operation group, were surgically ovariectomized. Specifically, after 1 week of acclimation, mice were subjected to a bilateral ovary removal or sham surgery by using sterilized surgical equipment under anesthesia. Menopausal dyslipidemia was induced in the female mice for 6 weeks. After induction of dyslipidemia, freeze‐dried FPE‐FGE (1:9, 3:7, and 9:1) was orally gavaged and administered once daily for 8 weeks. The 1× mouse dose (5.2 mg/kg) was used to evaluate the anti‐dyslipidemic effects, following the method of a previous study (Ha et al., 2023).

### 2.5 Blood chemistry tests

For the determination of the safety‐related biomarkers aspartate aminotransferase (AST), alanine aminotransferase (ALT), and blood urea nitrogen (BUN), and the blood lipids total cholesterol (TC), HDL‐cholesterol (HDL‐C), LDL‐cholesterol (LDL‐C), and triglyceride (TG), plasma samples were transferred to an automatic biochemical analyzer (Accute BA™, Toshiba Corp., Tokyo, Japan). Creatinine (CRE) was measured using a commercial kit (#80350, Crystal Chem, Inc., Elk Grove Village, IL, USA).

### 2.6 Immunoblotting

To determine estrogen receptor α (ERα) and estrogen receptor β (ERβ), uterus tissue was homogenized by Kimble® vortex mixer (DWK Life Sciences, Millville, NJ, USA), following the method of a previous study (Ha et al., 2023). Electrophoresed proteins were transferred to a membrane, blocked with 5% nonfat dried‐milk proteins, and probed with the corresponding antibodies against ERα [sc‐8002 (1:1000), Santa Cruz Biotechnology, Inc., Dallas, TX, USA], ERβ [sc‐390243 (1:1000), Santa Cruz Biotechnology, Inc.]. The membrane was incubated with horseradish peroxidase‐conjugated secondary antibodies. Proteins were visualized with an enhanced chemiluminescence system (ImageQuant™ LAS 500, Thermo Fisher Scientific Inc., Waltham, MA, USA).

### 2.7 Enzyme‐linked immunosorbent assay (ELISA)

For the determination of E2, LH, and FSH, whole blood was drawn from the abdominal aorta and inferior vena cava, collected in anticoagulant tubes (Becton, Dickinson and Company [BD], Franklin Lakes, NJ, USA), centrifuged, and plasma samples collected, following the method of a previous study (Ha et al., 2023). In addition, for the determination of eNOS‐NO‐cGMP signaling‐pathway‐related biomarkers eNOS, cGMP, and PKG, the abdominal aorta tissue was lysed according to the enzyme‐linked immunosorbent (ELISA) kit supplier's protocol. The levels of biomarkers were assayed using commercial ELISA kits as follows: E2 (CSB‐E05109m; Cusabio Technology LLC, Houston, TX, USA), LH (CSB‐E12770m; Cusabio Technology LLC), FSH (CSB‐E06871m; Cusabio Technology LLC), eNOS (ab230938; Abcam, Cambridge, UK), cGMP (ab133052; Abcam), and PKG (LS‐F9645; LifeSpan BioSciences Inc. [LS Bio], Shirley, MA, USA).

### 2.8 Modified diazotization assay using the Griess method

To determine NO, plasma samples were transferred to a 96‐well plate, induced by adding sulfanilamide into the reaction buffer, and incubated for 10 min at room temperature. Then the final reaction was activated by adding naphthyl ethylenediamine to the stabilizer buffer and incubated for 10 min at room temperature. The concentration of NO was measured using a commercial kit (21023; iNtRON Biotechnology Inc., Seongnam, Korea).

### 2.9 Statistical analysis

All experimental values are reported as the mean ± SD using GraphPad Prism 9 (GraphPad Software, Inc., San Diego, CA, USA). Significant differences were analyzed using a one‐way analysis of variance (ANOVA) followed by Dunnett's post‐hoc tests (*p* < .05).

## 3 RESULTS AND DISCUSSION

### 3.1 Fermentation of *Pueraria lobata* root or *Glycine max* (*L.*) Merrill extracts

Food‐origin lactic acid bacteria are characterized by high intestinal reach and settlement due to their high acid resistance and bile resistance (Chang et al., 2010; Jung et al., 2019). Therefore, we isolated 56 LABs from fermented food (Ha et al., 2023) and inoculated them into *Pueraria lobata* root or *Glycine max* (*L*.) Merrill extracts. After fermentation, we evaluated the concentrations of glycosides or aglycones such as daidzein and genistein (data not shown).

**FIGURE 1:**
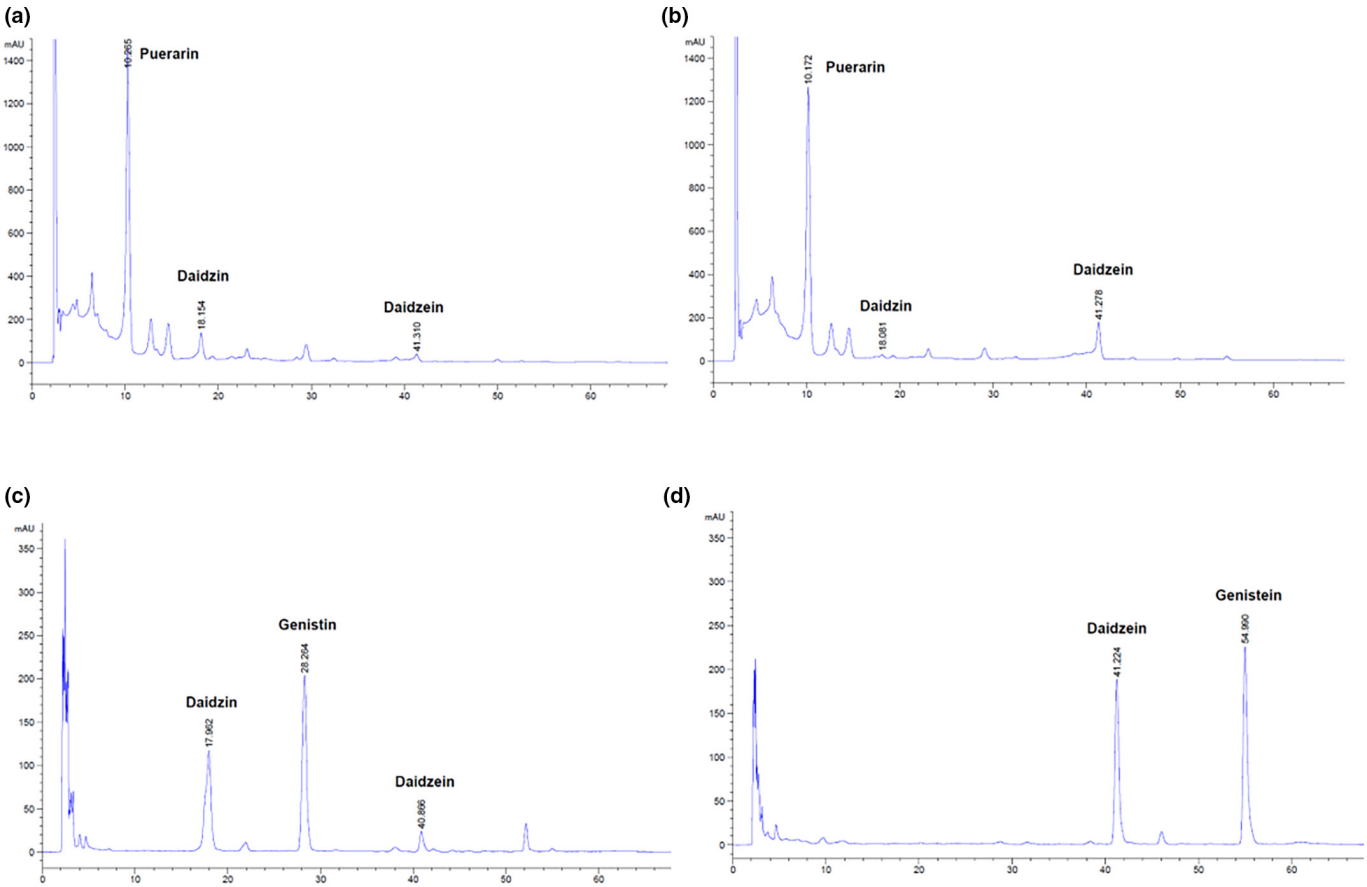
Analysis of changes in representative substances after fermentation. *Pueraria lobata* root extract (a); fermented *P. lobata* root extract (b); *Glycine max* (*L*.) Merrill extract (c); fermented *Glycine max* (*L*.) Merrill extract (d).

Among these, *L. plantarum* CCHR1 (KCCM13337P) was selected as a suitable strain for *Pueraria lobata* root extract and *L. plantarum* CCHR2 (KCCM13338P) was found suitable for *Glycine max* (*L*.) Merrill extract. No special substances were detected in the FPE (Figure 1a,b), and the content of daidzein and genistein increased noticeably in the FGE (Figure 1c,d).

These results suggest that food‐origin lactic acid bacteria have relatively good growth potential in the external environment and thus good fermentation ability for each natural product.

### 3.2 Effects of administering FPE‐FGE on the safety in OVX mice

**FIGURE 2:**
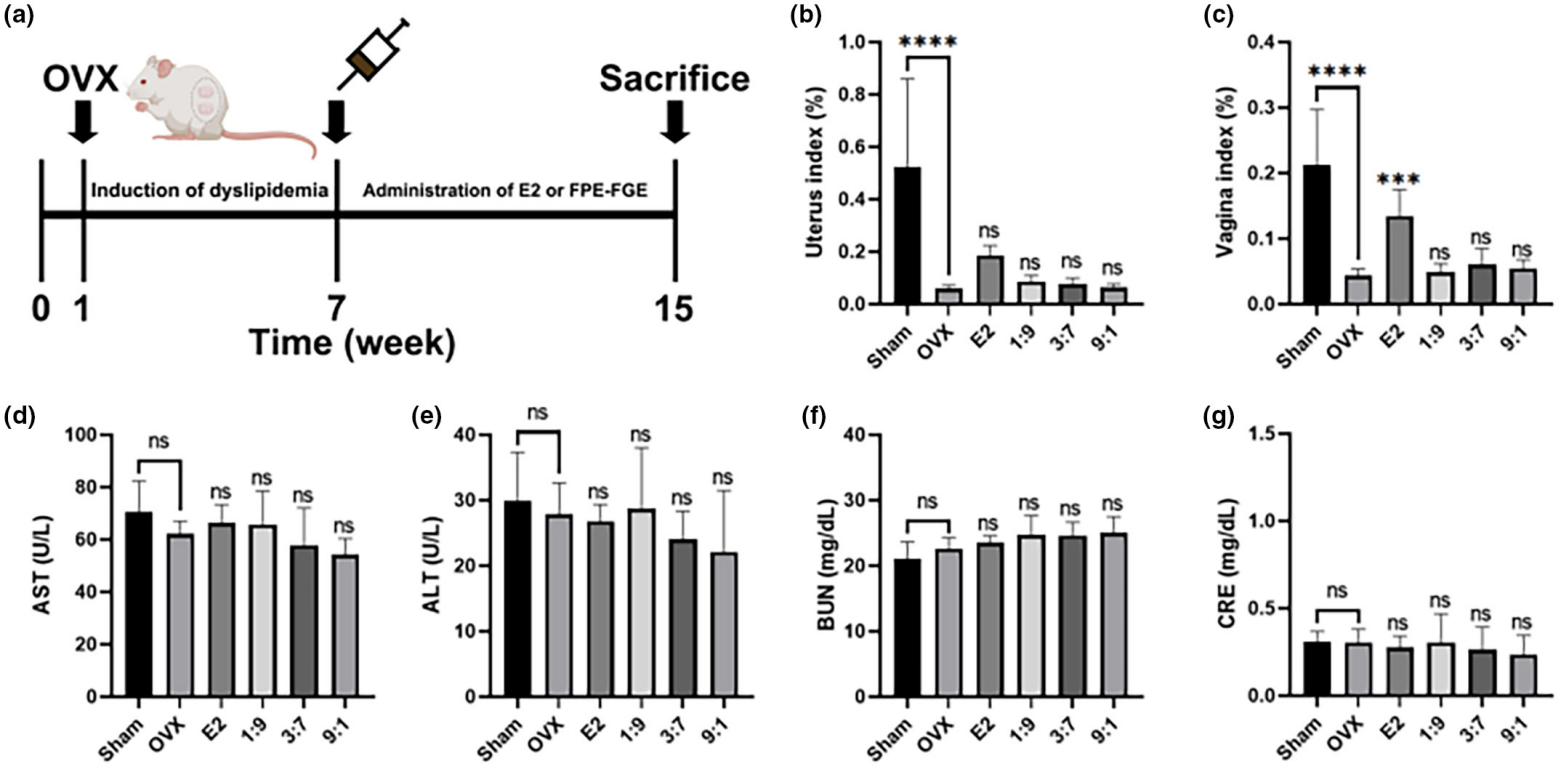
Safety of the administration of FPE‐FGE (1:9, 3:7, and 9:1) in OVX mice. Experimental design, (a); effects on the uterus index (%) (b); vagina index (%) (c); aspartate aminotransferase (AST) (d); alanine aminotransferase (ALT) (e); blood urea nitrogen (BUN) (f); and creatinine (CRE) (g). Values are indicated as mean ± SD (*n* = 7). *p < .05, **p < .01, ***p < .001, and ****p < .0001 compared with the OVX group. FPE‐FGE, mixture of fermented *Pueraria lobata* root and *Glycine max* (*L*.) Merrill extracts; OVX, ovariectomized.

The Republic of Korea's Ministries of Food and Drug Safety (Korea MFDS) presents safety evaluation indicators through the guidelines for functional foods. On the basis of this, FPE‐FGE (3:7) did not affect the safety‐related biomarkers, such as uterus index (%), vagina index (%), AST, ALT, BUN, and CRE. Therefore, to further investigate the safety of FPE‐FGE (1:9, 3:7, and 9:1) in vivo by oral administration to OVX mice daily for 8 weeks, we evaluated the uterus index (%), vagina index (%), AST, ALT, BUN, and CRE (Figure 2a). The OVX surgery induced severe uterine and vaginal atrophy, resulting in the reduction of uterus index (%) and vagina index (%) to 88.4% and 79.6%. The intraperitoneal injection of E2 (10 μg/kg) inhibited OVX‐induced uterine and vaginal atrophy to 209.2% and 210.3%. However, orally administration of FPE‐FGE (1:9, 3:7, and 9:1) were not significant (Figure 2b,c).

Next, we measured the liver function, including the plasma levels of AST and ALT, and kidney function, including the plasma levels of BUN and CRE. No significant difference was found in E2 (10 μg/kg) or FPE‐FGE (1:9, 3:7, and 9:1) (Figure 2d–g). These results suggest that FPE‐FGE (1:9, 3:7, and 9:1) were safe functional food that does not affect liver and kidney function.

### 3.3 Phytoestrogenic effects of administering FPE‐FGE in OVX mice

**FIGURE 3:**
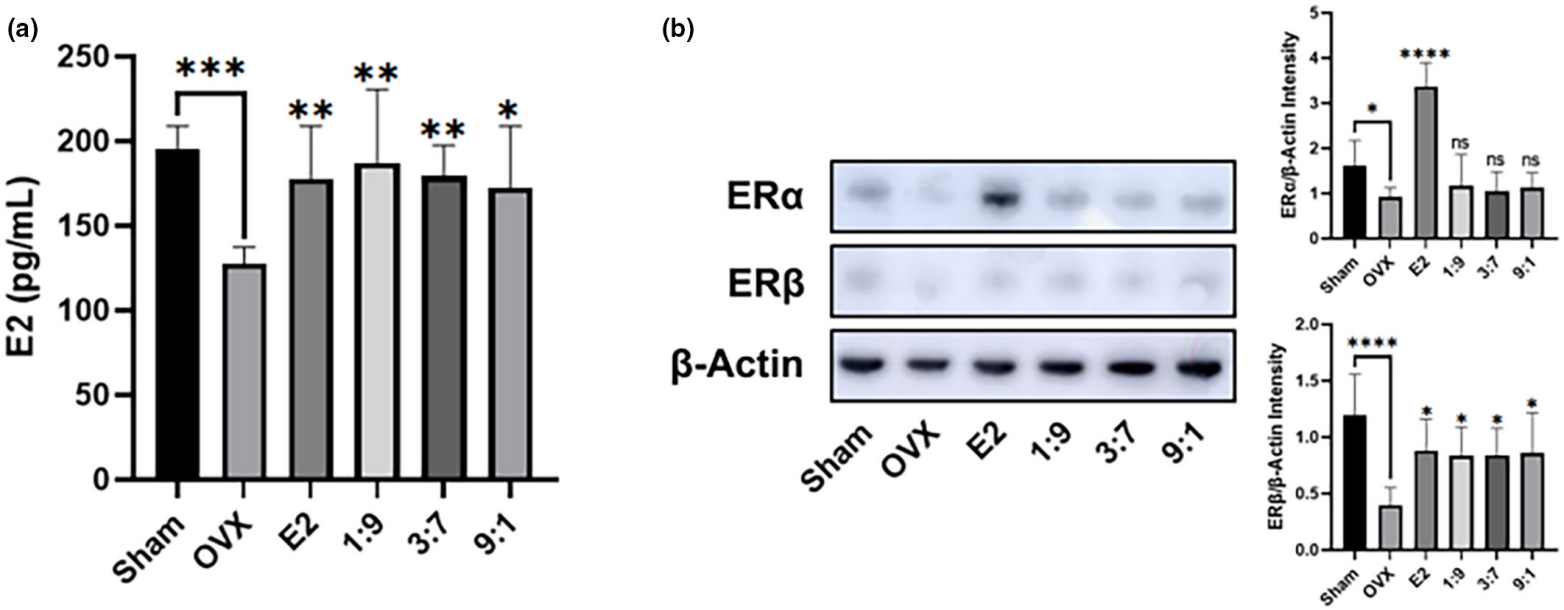
Phytoestrogenic effects of the oral administration of FPE‐FGE (1:9, 3:7, and 9:1) in OVX mice. Effects on 17β‐estradiol (E2) (a), expression of estrogen receptor α (ERα) and ERβ (b). Values are indicated as mean ± SD (*n* = 7). *p < .05, **p < .01, ***p < .001, and ****p < .0001 compared with the OVX group. FPE‐FGE, a mixture of fermented *Pueraria lobata* root and *Glycine max* (*L*.) Merrill extracts; OVX, ovariectomized.

To evaluate the phytoestrogenic effects of FPE‐FGE (1:9, 3:7, and 9:1) in OVX mice, we measured the plasma levels of E2 and the expression of uterus ERα and ERβ. The OVX surgery induced a reduction of E2 levels to 34.4%. Intraperitoneal injection of E2 (10 μg/kg) or oral administration of FPE‐FGE (1:9, 3:7, and 9:1) increased the OVX‐induced reduction of E2 to 39.4% or 46.7%, 40.6%, and 35.0% (Figure 3a).

Next, we measured the expression of uterus ERα and ERβ. The OVX surgery reduced ERα and ERβ expression to 43.0% and 66.7%. However, intraperitoneal injection of E2 (10 μg/kg) inhibited the OVX‐induced reduction of ERα and ERβ expression to 266.4% and 120.9%. However, oral administration of FPE‐FGE (1:9, 3:7, and 9:1) did not affect the expression of ERα. In contrast, oral administration of FPE‐FGE (1:9, 3:7, and 9:1) increased the expression of ERβ to 109.5%, 110.1%, and 115.3% (Figure 3b).

Women experiencing menopause may have various physiological responses, which can lead to dyslipidemia, hot flashes, sleep problems, depression, and osteoporosis. These symptoms begin with decreased E2 levels (Minkin, 2019). The transmission of E2 signals through estrogen receptors, such as ERα and ERβ, is essential to women's genital function (Pastore et al., 2012). However, ERα is frequently mutated in breast cancer and linked to ligand‐independent growth and metastasis (Li et al., 2022). In addition, breast cancer is known to be catalyzed by estrogen and is associated with the expression of ERα (Anbalagan & Rowan, 2015). In contrast, ERβ inhibits breast cancer cell migration and invasion (Song et al., 2019). In addition, ERβ inhibits the anti‐proliferative functions in ERα‐positive cells, such as MCF‐7 cells (Hapangama et al., 2015). Therefore, downregulating the expression of ERα and upregulating the expression of ERβ is a beneficial strategy for preventing breast cancer.

### 3.4 Effects of administering FPE‐FGE on OVX mice's gonadotropin levels

**FIGURE 4:**
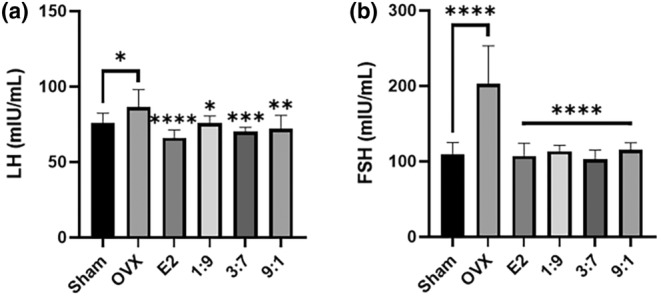
Effects of the oral administration of FPE‐FGE (1:9, 3:7, and 9:1) on gonadotropin levels in OVX mice. Effects on luteinizing hormone (LH) (a) and follicle‐stimulating hormone (FSH) (b). Values are indicated as mean ± SD (*n* = 7). *p < .05, **p < .01, ***p < .001, and ****p < .0001 compared with the OVX group. FPE‐FGE, mixture of fermented *Pueraria lobata* root and *Glycine max* (*L*.) Merrill extracts; OVX, ovariectomized.

To investigate whether oral administration of FPE‐FGE (1:9, 3:7, and 9:1) could inhibit LH and FSH in OVX mice, we measured the plasma levels of LH and FSH. The OVX surgery induced an increase in LH and FSH to 14.2% and 86.4%. However, intraperitoneal injection of E2 (10 μg/kg) inhibited the increase in LH and FSH to 23.7% and 47.2%. In addition, oral administration of FPE‐FGE (1:9, 3:7, and 9:1) inhibited the increase of LH to 12.4%, 18.9%, and 16.9% and FSH to 44.1%, 49.2%, and 42.9% (Figure 4a,b).

Menopause in women induces a reduction of E2, resulting in increased gonadotropin levels, including LH and FSH (Kim et al., 2014; Luderer & Schwartz, 1994). Accordingly, elevated LH and FSH levels are anticipated in OVX mice (Luderer & Schwartz, 1994). Gonadotropins are essential to reproductive health; however, elevated LH and FSH levels contribute to the acceleration of dyslipidemia by inhibiting NO synthesis (Meng et al., 2019); NO inhibits atherosclerosis by improving vasomotor dysfunction (Raman et al., 2011; Tentolouris et al., 2000). Therefore, to understand the anti‐dyslipidemia effects of the oral administration of FPE‐FGE (1:9, 3:7, and 9:1) on OVX mice, the inhibitory effects of gonadotropins or inducible effects of the eNOS‐NO‐cGMP signaling pathway‐related biomarkers have been examined.

### 3.5 Effects of administering FPE‐FGE on OVX‐induced dyslipidemia in mice

**FIGURE 5:**
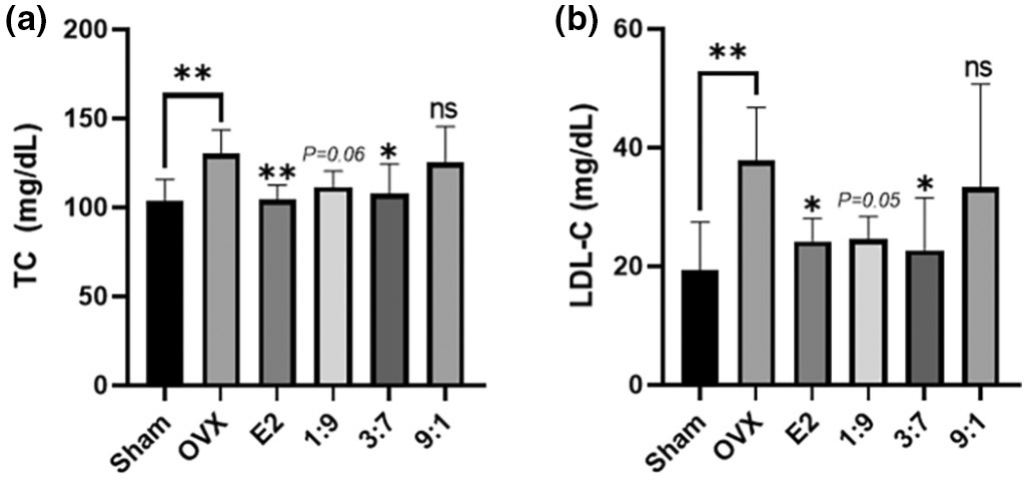
Anti‐dyslipidemic effects of the oral administration of FPE‐FGE (1:9, 3:7, and 9:1) in OVX mice. Effects on total cholesterol (TC) (a) and LDL‐cholesterol (LDL‐C) (b). Values are indicated as mean ± SD (*n* = 7). *p < .05, **p < .01, ***p < .001, and ****p < .0001 compared with the OVX group. FPE‐FGE, a mixture of fermented *Pueraria lobata* root and *Glycine max* (*L*.) Merrill extracts; OVX, ovariectomized.

Next, we measured the plasma levels of TC, HDL‐C, LDL‐C, and TG to evaluate the curative effects of FPE‐FGE (1:9, 3:7, and 9:1) on OVX‐induced dyslipidemia in mice. The OVX surgery increased the TC and LDL‐C to 25.5%, 94.9%. However, the intraperitoneal injection of E2 (10 μg/kg) or oral administration of FPE‐FGE (3:7) repressed an increase in TC and LDL‐C (Figure 5a,b). However, the HDL‐C and TG had no significant effect (data not shown).

### 3.6 Effects of administering FPE‐FGE on OVX mice's eNOS‐NO‐cGMP signaling pathway

Next, to investigate the mechanism of curative effects of FPE‐FGE (3:7), we measured the eNOS‐NO‐cGMP signaling pathway‐related biomarkers, including eNOS, NO, cGMP, and PKG. Increased expression of eNOS in endothelial cells also increases the occurrence of NO, which in turn increases the expression of cGMP in smooth muscle cells. This pathway eventually induces vasodilation and strengthens blood circulation (Cho et al., 2018).

**FIGURE 6:**
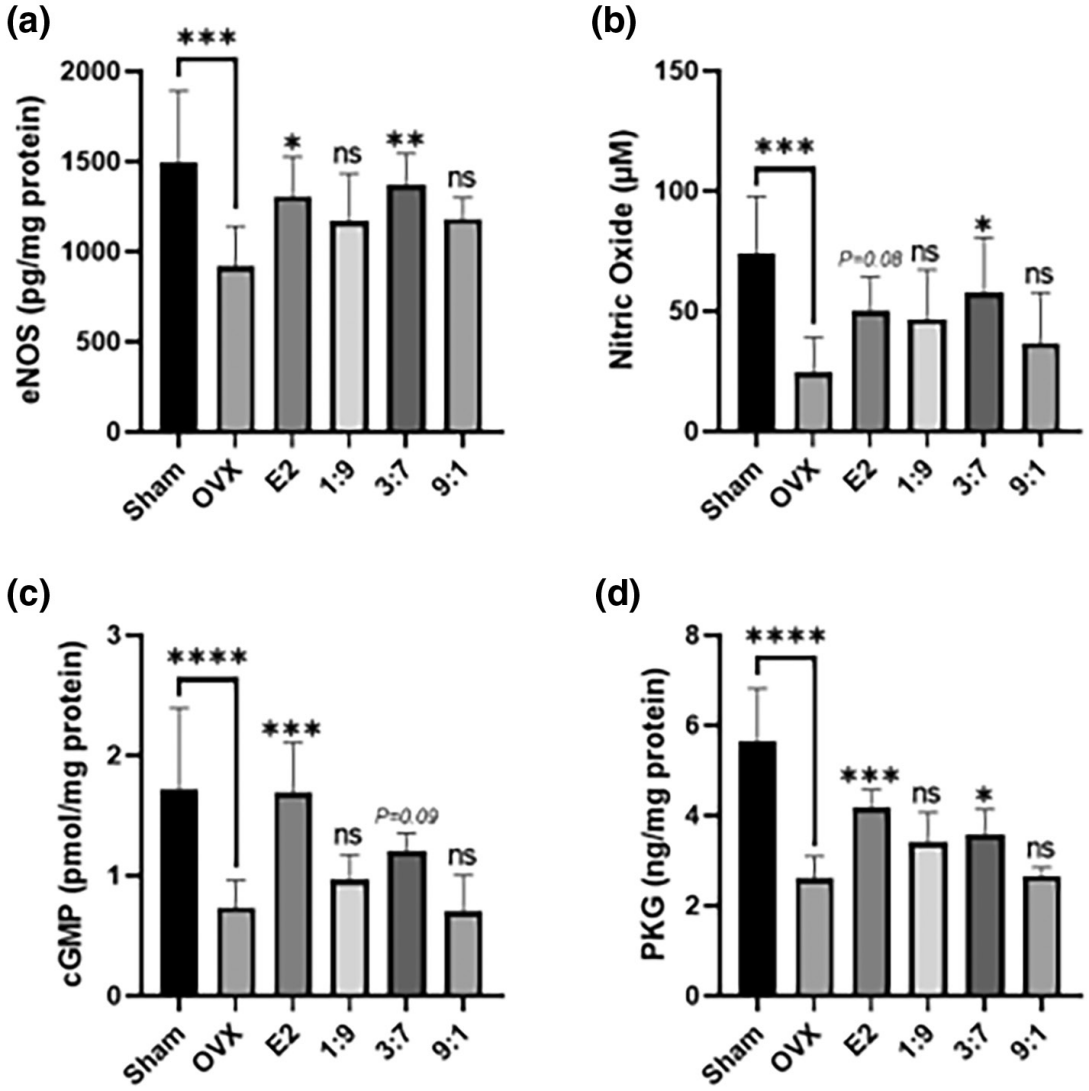
Effects of the oral administration of FPE‐FGE (1:9, 3:7, and 9:1) on nitric oxide synthase‐nitric oxide‐cyclic guanosine monophosphate (eNOS‐NO‐cGMP) signaling pathway in OVX mice. Effects on endothelial nitric oxide synthase (eNOS) (a), nitric oxide (NO) (b), cyclic guanosine monophosphate (cGMP) (c), and protein kinase G (PKG) (d). Values are indicated as mean ± SD (*n* = 7). *p < .05, **p < .01, ***p < .001, and ****p < .0001 compared with the OVX group. FPE‐FGE, a mixture of fermented *Pueraria lobata* root and *Glycine max* (*L*.) Merrill extracts; OVX, ovariectomized.

The OVX surgery induced a reduction of the abdominal aortic eNOS, cGMP PKG to 38.7%, 57.4%, 53.9%, and plasma levels of NO to 66.6%. However, intraperitoneal injection of E2 (10 μg/kg) inhibited the reduction of eNOS, NO, cGMP, and PKG to 42.6%, 104.1%, 131.3%, and 61.3%. Interestingly, oral administration of FPE‐FGE (3:7) inhibited the reduction of eNOS, NO, cGMP, and PKG to 49.1%, 133.9%, 63.7%, and 37.9%. However, oral administration of FPE‐FGE (1:9 and 9:1) had no significant effect (Figure 6a–d). These results suggest that FPE‐FGE (3:7), which includes a combination of various glycosides, such as puerarin, daidzin, and genistin, and aglycones, such as daidzein and genistein, additively or synergistically, can reduce the levels of blood lipids by upregulating eNOS‐NO‐cGMP signaling pathway.

Flavonoids are known to enhance the production of NO, causing endothelium‐dependent vasorelaxation in the rat aorta (Benito et al., 2002). However, there were no studies that confirmed the change in the eNOS‐NO‐cGMP signaling pathway according to the ratio of glycosides and nonglycosides among isoflavones. This study is the first study to investigate the eNOS‐NO‐cGMP signaling pathway according to the ratio of glycosides and aglycones, and it has been proven that a mixture of glycosides and aglycones ratio (3:7) is the most effective in improving the cardiovascular system.

## 4 CONCLUSION

Oral administration of FPE‐FGE (1:9, 3:7, and 9:1) increased the levels of E2 and the expression of uterus ERβ but did not significantly affect the expression of uterus ERα. Moreover, FPE‐FGE (1:9, 3:7, and 9:1) reduced the levels of gonadotropins, such as LH and FSH. These results suggest that FPE‐FGE (1:9, 3:7, and 9:1) can exert phytoestrogenic effects by increasing the levels of E2 through the expression of uterus ERβ. Especially, FPE‐FGE (3:7), which includes a combination of various glycosides or aglycones, such as daidzein and genistein, additively or synergistically attenuated the menopausal dyslipidemia by upregulating eNOS‐NO‐cGMP signaling pathway. Although there is a limitation that there are no clinical trials, there are sufficient grounds to move on to clinical trials because significant results have been obtained in animal experiments.

In conclusion, combinations of various phytoestrogens can exert phytoestrogenic effects in women experiencing menopause with dyslipidemia through the OVX mice. If further clinical trials confirm the same symptom improvement, it could become potentially commercial functional foods for improving menopausal symptoms.

## AUTHOR CONTRIBUTIONS

**Insuk Choi:** Conceptualization (lead); resources (lead). **Hyo‐Min Jang:** Methodology (equal); writing – original draft (lead). **Jimyeong Ha:** Methodology (equal); visualization (equal); writing – review and editing (lead).

## FUNDING INFORMATION

This research was supported by the CMG Pharmaceutical Co., Ltd. and the CHA Advanced Research Institute (CARI‐RN‐84).

## CONFLICT OF INTEREST STATEMENT

The authors declare that they have no competing interests.

## Data Availability

The data that support the findings of this study are available on request from the corresponding author.
